# Effects of Heat Stress in Dairy Cows Raised in the Confined System: A Scientometric Review

**DOI:** 10.3390/ani13030350

**Published:** 2023-01-19

**Authors:** Karen Dal’ Magro Frigeri, Kariane Donatti Kachinski, Nédia de Castilhos Ghisi, Matheus Deniz, Flávio Alves Damasceno, Matteo Barbari, Piotr Herbut, Frederico Márcio Corrêa Vieira

**Affiliations:** 1 Biometeorology Study Group (GEBIOMET), Federal University of Technology—Paraná (UTFPR), Dois Vizinhos 85660-000, Brazil; 2 Graduate Program in Biotechnology (PPGBIOTEC), Campus Ponta Grossa (UTFPR-PG), Federal University of Technology—Paraná, Ponta Grossa 84017-220, Brazil; 3 Graduate Program in Biotechnology (PPGBIOTEC), Campus Dois Vizinhos (UTFPR-DV), Federal University of Technology—Paraná, Dois Vizinhos 85660-000, Brazil; 4 School of Veterinary Medicine and Animal Science, São Paulo State University, Botucatu 18618-681, Brazil; 5 Department of Engineering, Federal University of Lavras (UFLA), Lavras 37203-202, Brazil; 6 Department of Agriculture, Food, Environment and Forestry, University of Florence, 50145 Florence, Italy; 7 Department of Rural Building, Faculty of Environmental Engineering and Land Surveying, The University of Agriculture in Krakow, 31-120 Kraków, Poland

**Keywords:** cattle, compost barn, free-stall, systematic review, tie-stall, biometeorology, CiteSpace

## Abstract

**Simple Summary:**

Studies on the effect of thermal stress on lactating cows have increased considerably in recent years. Feedlot systems for dairy cows have become popular around the world. This article aims to develop a scientometric analysis to evaluate studies on thermal stress in lactating cows housed in a free-stall, tie-stall, and compost-bedded pack barn system. A total of 604 studies from the Web of Science database was considered for this study. The data obtained from the Web of Science was exported to Citespace software. The most used keywords by the researchers were “heat stress”, “dairy cow” and “cattle”. The most relevant countries, authors, institutions, and networks of co-occurrences for our research were highlighted. This article provides a comprehensive review of thermal stress in lactating cows housed in confinement in the last 22 years and contributes to future research in this area.

**Abstract:**

Due to climate change, heat stress is a growing problem for the dairy industry. Based on this, annual economic losses in the dairy sector are verified mainly on a large scale. Despite several publications on thermal stress in lactating dairy cows in confinement systems, there need to be published reviews addressing this issue systematically. Our objective was to scientometrically analyze the effects of heat stress in dairy cows managed in a confinement system. Based on PRISMA guidelines, research articles were identified, screened, and summarized based on inclusion criteria for heat stress in a confinement system. Data was obtained from the Web of Science. A total of 604 scientific articles published between 2000 and April 2022 were considered. Data was then analyzed using Microsoft Excel and CiteSpace. The results pointed to a significant increase in studies on heat stress in lactating cows housed in confinement systems. The main research areas were Agriculture, Dairy Animal Science and Veterinary Sciences. The USA showed the highest concentration of studies (31.12%), followed by China (14.90%). Emerging themes included heat stress and behavior. The most influential journals were the Journal of Dairy Science and the Journal of Animal Science. The top authors were L. H. Baumgard and R. J. Collier. The leading institutions were the Chinese Academy of Agricultural Sciences, followed by the State University System of Florida and the University of Florida. The study maps the significant research domains on heat stress of lactating cows in confinement systems, discusses implications and explanations and highlights emerging trends.

## 1. Introduction

Heat stress is a biophysical condition that directly impacts the biological system of dairy cows [1]. Thus, behavioral, physiological, and endocrine mechanisms are used to mitigate the effects of heat stress [2]. However, this increases the metabolic rate of the cow and consequently results in production losses [3,4,5]. Environmental conditions may result in insufficient mechanisms adopted for body heat dissipation by lactating cows. Thermal stress conditions result in acute and chronic responses [6,7,8]. The critical response originates from the endocrine and nervous systems’ homeostatic regulators, and the chronic response arises from the homeorrhetic regulators of the endocrine system [9]. Thus, both responses impact lactating cows’ metabolism, leading to body thermoregulation energy loss. 

Thermoregulation is essential for lactating cows to maintain homeostasis and homeothermia by balancing body heat gain and abatement [10]. Heat dissipation by lactating cows occurs mainly by heat and mass transfer mechanisms [11]; for example, sensible (conduction, convection, radiation) and latent (evaporation) heat exchange. Heat energy loss by evaporation is the only mechanism to reduce the animal’s internal temperature when the environmental temperature is higher than the body temperature [12]. Cows have a thermal equilibrium zone between the body and effective temperature dissipation. The thermoneutral zone corresponds to the temperature limits (lower and upper), in which the animal organism minimally mobilizes thermoregulatory mechanisms to control body temperature [13]. The environmental temperature for European lactating cows to remain thermoneutral ranges between −0.5 °C and 20 °C [14], while relative humidity ranges between 40% and 60% [15]. In addition to the thermoneutral zone, thermal comfort indices help interpret interactions between the environment and animal, resulting in various responses, which must be interpreted according to the animal species and individual characteristics. For example, the temperature and humidity index is the leading thermal comfort index used in animal production; however, a disadvantage is that it only considers air temperature and relative humidity variables [16]. So, there is a discussion about the ideal values of dairy cattle. The National Weather Service [17] estimates that THI up to 74 represents safe environments for animals; a range of 75 to 78 requires alertness; a range of 79 to 83 is dangerous for animals; and environments with THI above 84 represent severe heat stress for animals. However, Rosenberg et al. [18] highlighted that behavioral and productive changes would occur when THI rises above 72 units, which may require alertness. Moreover, Bohmanova et al. [19] and Collier et al. [20] identified production losses and changes in respiratory frequency and behavior with THI above 65 units; further, they defined a threshold of 68 THI units for cows with an average daily yield of 35 kg/day.

In addition to environmental temperature and RH content, wind speed and solar radiation compile the picture of the main thermal stressors. These factors drastically affect the animal production system due to their potential to alter the physiological state, health, and animal welfare [21]. Therefore, the latest Intergovernmental Report on Climate Change showed that Earth’s temperature has increased by 1.2 °C since the Industrial Revolution [22]. This increase in average temperature has contributed to the rise in the intensity of heat waves [23]. As a result, cows increasingly depend on strategies to alleviate heat stressors. To mitigate the effects of heat stress in lactating cows, confinement systems have emerged as an alternative to increase production, improve milk quality, ensure herd health, enable animal welfare conditions, provide better use of farming areas and, consequently, improve the thermal comfort of animals. Therefore, dairy cows’ housing systems should reduce animals’ exposure to environmental stressors to reduce production losses due to heat stress. Therefore, control of the indoor thermal environment in confinement systems is essential [24], and the use of a ventilation system and evaporative cooling should be well-planned [25] so that heterogeneity of the environment in these systems is avoided [26]. This is because the heterogeneity of the environment directly interferes with the thermoregulation of body temperature in dairy cows [27]. 

Heat stress in dairy cows has increasingly attracted the attention of researchers and professionals worldwide. We performed a systematic review using a scientometric analysis approach to gain insight into heat stress in dairy cows raised in confinement systems (e.g., compost barn, free-stall, and tie-stall). The scientometric analysis is a quantitative study method [28]. It is increasingly being applied in scientific domains as it is a reliable source of information [29]. This analysis consists of scientific mapping studies [30] revealing research trends in various knowledge areas [31]. Thus, our study proposed to (i) investigate the state of the art of research involving heat stress in lactating cows in confinement systems in the last 22 years (from January 2000 to April 2022); (ii) understand which themes journals publish more; (iii) identify the principal authors, institutions, and countries where the studies are developed; (iv) analyze the main keywords; and (v) identify future trends in this line of research.

## 2. Materials and Methods

### 2.1. Search Strategy 

This review was conducted according to the guidelines of the Preferred Reporting Items for Systematic Reviews and Meta-Analyses (PRISMA; Moher et al. [32]) protocols using a scientometric analysis approach. Systematic searches on “Heat stress in a dairy cow in confinement system” were performed on the Web of Science (WoS). The search terms used to search the WoS Core Collection were TS (Topic Search) = (dairy AND cow) AND (“heat stress” OR “thermal stress”) AND (“compost-bedded pack barn” OR “compost barn”) AND (free-stall OR “free stall”) AND (tie-stall OR “tie stall”). 

### 2.2. Study Inclusion Criteria and Screening

We selected studies on the effects of heat stress on lactating cows in a confinement system. Inclusion and exclusion criteria for the scientometric analysis were determined a priori. The WoS search returned 2245 scientific studies. Our study consisted of four stages. Stage 1: Studies written in all languages and document types (articles, review articles, early access, data articles, conference papers and meeting abstracts) were included. Step 2: Studies published before the year 2000 were excluded due to our objective of identifying the effects of heat stress on lactating cows in a confinement system published between January 2000 and April 2022. The remaining studies were reviewed in step 3. Step 3: Study titles and abstracts were evaluated to identify and remove studies that did not use lactating cows (e.g., studies that dealt with dry cows and heifers), confinement systems (e.g., studies that dealt with cows on a pasture-based system) and heat stress (e.g., studies that dealt with cold heat stress). Step 4: Finally, the completed studies were read in detail. Studies that did not address the effect of heat stress on lactating cows in a confinement system were excluded. The remaining studies (*n* = 604) were included in the scientometric analysis. 

### 2.3. Data Extraction

The Analyze Results tool of WoS was used to quantify the types of documents, authors, categories, areas of publications, languages, and countries. The Citation Report tool was used to determine the average H-index, the total number of citations per year, publications and the 10 most cited articles. 

### 2.4. Scientometric Analysis Methods

**Figure 1:**
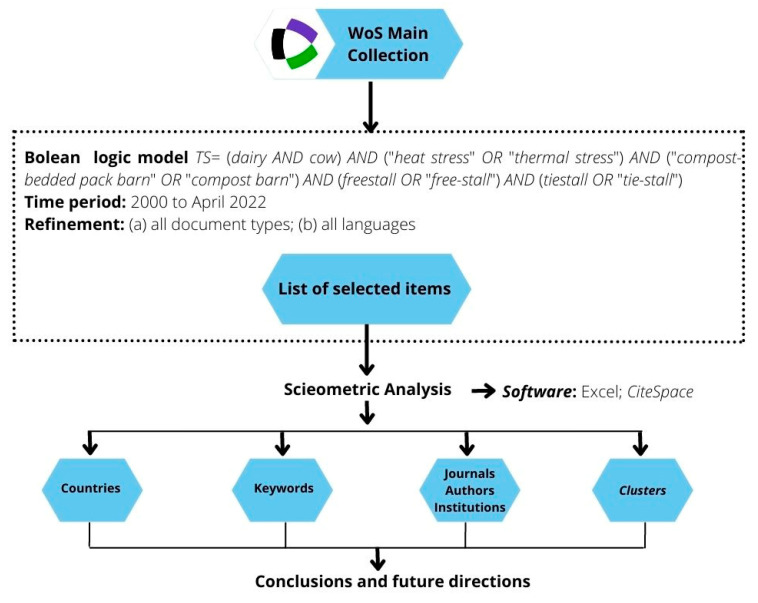
Research design.

The data acquisition method and scientometric analysis framework for this study are shown in Figure 1. The selected studies’ metadata (e.g., title, abstract and cited references) was exported to CiteSpace (version 6.0 R1). CiteSpace is free scientometric software that allows one to explore a given area of knowledge and assess the main parameters involved through co-citation network analysis and graphical visualization [33]. 

Two scientometric techniques were performed in this study: (1) co-occurrence analysis of countries, institutions, and keywords; (2) cluster analysis performed based on co-cited documents of countries and keywords. The clusters were ranked by weight. CiteSpace gives a zero identification (#0) to the cluster with the highest number of members. The modularity Q and the average silhouette were determined in each generated co-occurrence network and the Clusters. The modularity Q-score measures the extent to which a network can be divided into independent components. The modularity score ranges from 0.000 to 1.000, with a value of 1.000 as a perfect representation [34]. The silhouette reflects the degree of homogeneity [35] and the uncertainties involved in interpreting a cluster [34]. The silhouette value ranges from −1.000 to 1.000. A value of 1.000 is the perfect representation [34]. 

CiteSpace showed the results infographically. The node represents the investigated item (e.g., Countries), and the links (lines) are the connections between the nodes. The nodes and links had different colors, indicating a chronological order. Thus, colder colors (for example, blue) represented older studies, and warmer colors (for example, yellow) represented more recent studies [33]. In addition, the software reported the Burst between the scientometric techniques. Burst indicates a more active research area or an emerging trend attracting attention to its scientific community [33]. Regarding the keyword cloud, the 25 words with the highest frequency among the studies were used. 

With the identification of key cards by CiteSpace, the 25 words that presented the highest frequencies were selected. These words were sequentially exported to the infogram program for word cloud development.

## 3. Results and Discussion

### 3.1. Publication Characteristics

**Figure 2:**
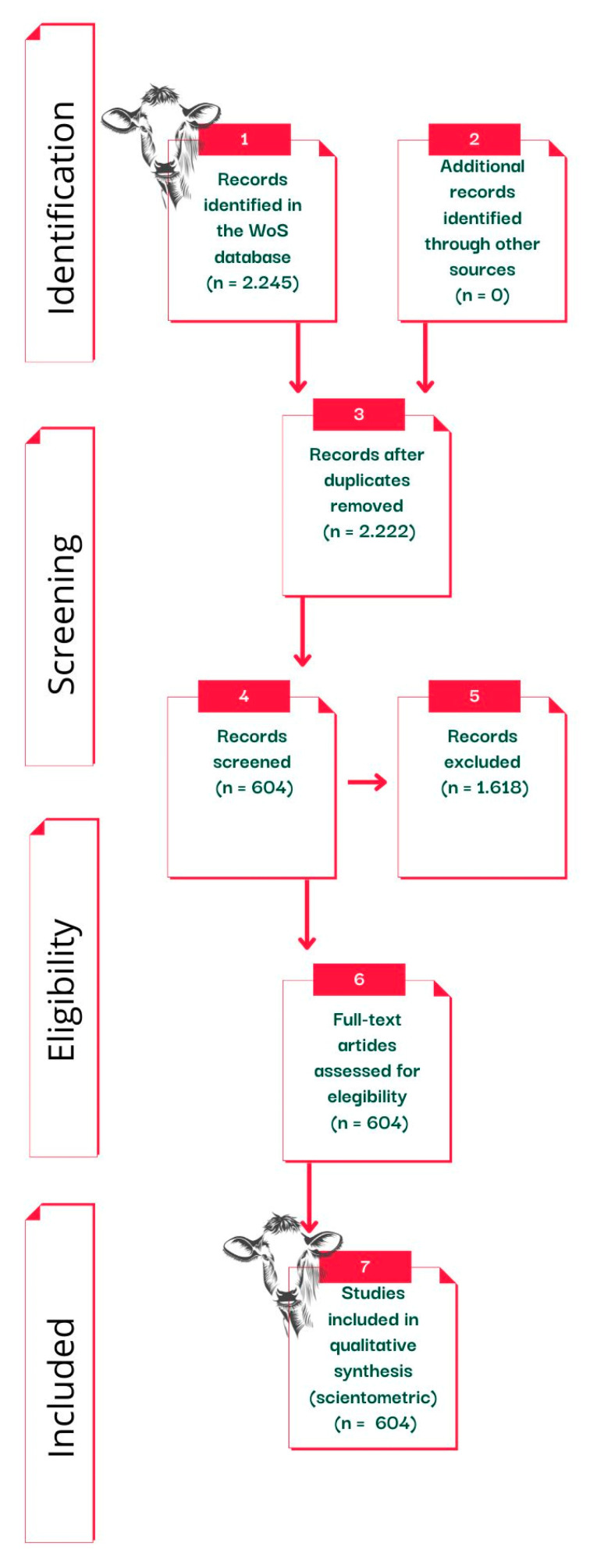
Flowchart following PRISMA guidelines (Moher et al. [32]), showing the total number of publications identified and the number of publications filtered at each stage of the selection process from the systematic review.

Of the 604 studies included in this review after the 4-step screening and assessment process (Figure 2), 82.11% were articles, followed by meeting abstracts (7.78%) and reviews (6.65%), conference papers (4.96%), early access (0.66%) and data articles (0.16%). English was the language used in 97.84% of the publications analyzed, followed by German (0.99%), Hungarian (0.33%), Spanish (0.33%), Polish (0.16%), Portuguese (0.16%) and Russian (0.16%). This fact confirms why English is considered science’s “mother language” [36]. The total number of citations found was 12.416. Thus, it is possible to determine the average proportion of citations per the study as 20.56, and the H-index of the publications was 49. The H-index measures the impact of scientific research, author, or scientific area [37]. Thus, among the publications evaluated for this review, 49 studies received at least 49 citations.

**Figure 3:**
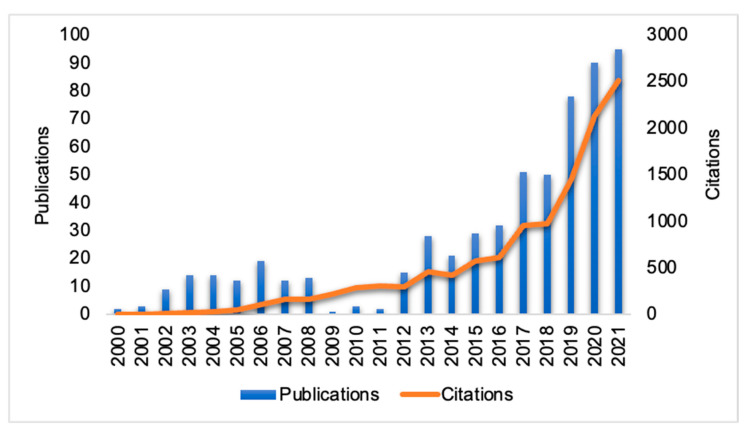
Relationship between the number of publications and citations per year of publications related to heat stress in dairy cows in a confinement system.

The publication year of the 604 studies included in this review ranged from 2000 to 2022. There has been an increase in the number of publications and citations about the topic of this study over the past 10 years (Figure 3). However, 87% of the studies were publications from 2021 to the present (2000–2010 = 3% and 2010–2020 = 10%). Two factors may have contributed to this: firstly, the emergence of the compost barn system in the 1980s (Virginia—USA, Janni et al. [38]) and second, the impacts of climate change [22], which contributed to decreased rainfall and increased duration and intensity of heat waves [23]. 

The compost barn confinement system aims to increase cow comfort, improve longevity, and reduce initial installation costs [39]. Thus, in the last decade, this confinement system has attracted substantial global interest [40,41], which has contributed to the considerable increase of research in this study area, in which studies have been reported in the United States [38,42,43], Italy [44,45,46], Spain [47], Germany [48], the Netherlands [49], Denmark [50], Brazil [26,51,52,53,54,55] and Australia [48].

The increase in global temperature (approximately 1.2 °C) has decreased the time cattle remain in thermal comfort zones, affecting the biological system and life of dairy cows [56]. The prolonged period of hot weather has been causing heat waves that have impacted dairy farming in some European countries [57,58]. The heat waves between the years 2003 and 2006 in France (between 1 to 5 August 2003, the temperature went from 25 °C to 37 °C and remained there until August 16, while in 2006, the heat waves were witnessed between 10 to 28 June with a temperature above 30 °C) contributed to the considered increase in dairy cow mortality from 12% to 24% [58]. In another study conducted in Italy between 2002 and 2007, Vitali et al. [57] highlighted the mortality of 46.610 dairy cows over 24 months of age caused by heat waves (temperature over 31 °C, relative humidity over 86% for six consecutive days). In addition, Vitali et al. [57] reported that the current and future climate scenario is expected to contribute to an increase in hot days (from 3 to 11 days), with an increase in the frequency (four or more events per year) and intensity of heat waves (temperature greater than 30 °C) and, that strategies should be implemented to be able to reduce the impacts of hot days. In addition, they reported that structural modifications to the facilities are needed, in addition, to feed management and the selection of more heat-resistant cows. Heat waves (3 days with a temperature of 32 °C or higher) were also witnessed in Canada between 2010 and 2012 [59]. The authors report that heat waves threaten the Canadian dairy industry because they have contributed to an increase in the mortality of 27% of dairy cows during this period.

Climatic extremes may intensify the problems of animals exposed to thermal challenges. Manica et al. [60], when analyzing heat wave events from 1981 to 2021, highlighted that Brazil had a 60% increase in the number of days with heat waves in the 2010s compared to the 2000s. As a response to heat waves, the authors reported that dairy cows housed in free-stalls showed an increase in respiratory rate (>80 mov. /min.) during the first four days of the prolonged heat waves (e.g., heat waves of more than five days). In contrast, milk production decreased from the fifth day of exposure to heat waves. In the United States, high temperatures (>40 °C) registered in 2022 promoted several cattle deaths in Kansas. In addition, extreme heat was recorded in Oklahoma, Texas, and other states [61].

### 3.2. Co-Country/Territory Analysis

**Figure 4:**
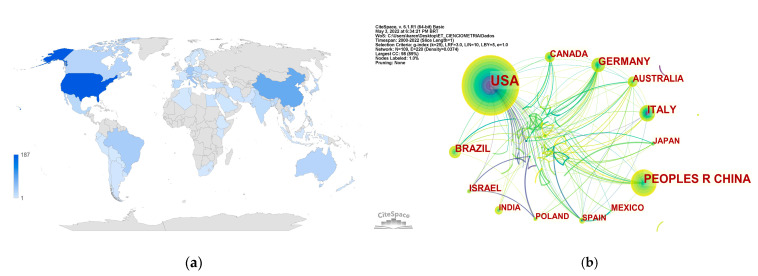
Geolocation of the 604 studies included in the review. (**a**) The geographic distribution of the studies is related to the color of each country, proportional to the number of publications (for interpretation purposes, the scale range from light blue which presents countries with the lowest number of publications to dark blue which presents countries with the highest number of publications, and gray which presents countries no publication). (**b**) The co-occurrence network of the nations is related by the node’s size, determined by the number of times that word was used and the links in the relationship between the countries.

Regarding the number of publications on heat stress in dairy cows in confinement, Figure 4a categorizes the leading countries. Table S1 presents the numerical data regarding these publications by country. It can be observed that the USA showed the highest percentage of publications (31.12%), followed by China (14.90%), Italy (7.61%), Germany (7.11%), Brazil (6.29%), Israel (4.47%), Australia (4.30%), Canada (4.30%), Mexico (3.47%), Japan (2.98%), Spain (2.98%), India (2.98%) and Poland (2.81%). 

Figure 4b shows the cooperation network between countries, whose generated network contained 72 nodes and 812 links. The nodes represent the countries, and the links represent their collaboration [62]. The modularity index score (Q) was 0.5357, and the average silhouette metric (S) was 0.8783. Silhouette measures average homogeneity [35], ranging from 1.000 to −1.000 [63]. The closer to 1000, the higher the reliability of the results [64]. Modularity, however, measures the extent to which a network can be broken down into multiple components or modules [33]. The silhouette found (0.8783) suggests consistency in our data. The size of the node informs the number of publications in each country; the thickness of the links indicates the intensity of cooperation between countries; and the colors of the links the time distance, represented in cold and warm tones (the coldest as the oldest publications and the warmest, the most recent) [33]. 

It is possible to see that the countries that presented the coldest colors (blue) in the links were the USA and Israel, indicating that these countries presented the older studies in research in this study area. Most of the studies with confinement and cooling systems for dairy cows were developed in the USA and Israel due to the moderate and severe heat stress in these countries, with air temperatures above 30°C and relative humidity of 40% [65]. This has contributed to developing studies in this line of research in these countries. This can also be proven because both countries have Burst, the USA (Burst = 8.00, 2001) and Israel (Burst = 3.83, 2006), as described in Table S1. Burst represents the increase in citations in a short period found in each country [31]. China, on the other hand, was the second country with the highest number of publications. Several studies on heat stress in the confinement system have been developed in China [66,67,68]. In a study designed in the same country, it was observed that cows in China presented heat stress between July and September (2014, 2015 and 2016), with a temperature and humidity index higher than 68 [8].

### 3.3. Publishing Areas

The top 10 areas of publications related to heat stress in dairy cows in confinement were Agriculture, Dairy and Animal Science (59.43%), followed by Veterinary Sciences (26.82%), Food Science Technology (20.36%), Zoology (8.11%), Reproductive Biology (7.78%), Agriculture Multidisciplinary (6.62%), Meteorology and Atmospheric Sciences (6.12%), Environmental Sciences (5.79%), Biophysics (5.13%) and Physiology (4.96%). This shows that these were the most active areas in this line of research between the years of 2000 and April 2022. Because this is a review evaluating the effect of heat stress in three confinement systems for dairy cows, the areas are highly related to the topic in our study. This information may help researchers choose possible areas for their future publications.

### 3.4. High-Cited Documents Analysis

The impact of publications is related to the number of times an article in a particular journal has been cited. Thus, it is interesting to observe which papers have the highest number of citations and which provide essential information about the impact of research on heat stress in lactating cows in confinement systems for the scientific community. It is important to note that, for this analysis, the number of citations does not refer to the number of WoS citations but the number of citations among the 604 documents retrieved for this work. We used the citation report function in WoS, and the results obtained are represented in Table 1, which shows the top 10 publications. These articles require reading for those who want to delve into the subject. 

The most cited publication is a literature review written by West [14] that depicts the effects of temperature, humidity, and solar radiation on milk production. In addition, the author described that environment modifications in confinement systems, including reducing radiation and using cooling systems, are crucial to increasing body heat loss in lactating cows. The author also portrays that using a negative pressure ventilation system in feedlots is a promising strategy. The demand for this system is growing among producers, consequently strongly attracting researchers to this study area. The author also details the use of genetic selection in the search for heat-tolerant cows. 

The second most cited article is also a literature review authored by Kadzere et al. [69] that addresses a holistic view of the factors that influence the incidence of heat stress in high-producing dairy cows. The authors portray that strategies are needed to alleviate metabolic and environmental heat loads in early lactation. New methods should be researched and developed, especially for high-producing cows in modern feedlots. 

The third most cited article was written by De Rensis and Scaramuzzi [70], which reports the impacts of heat stress on dairy cow reproduction. To mitigate the losses caused by heat stress on reproduction, the authors describe that using fans, sprinklers, or negative pressure systems is necessary, especially during the summer.

**Table 1:** Ranking of the most cited publications related to heat stress in dairy cows managed in confinement systems with title, scientific journal, year of publication, total citations, and average per year, according to the Web of Science (2022).

Ranking	Title	Journal	Year	Total Citations	Annual Citation Rate	Citation
1	Effects of heat stress on production in dairy cattle	Journal of Dairy Science	2003	912	45.6	West [14]
2	Heat stress in lactating dairy cows: a review	Livestock Production Science	2002	701	33.38	Kadzere et al. [69]
3	Heat stress and seasonal effects on reproduction in the dairy cow—a review	Theriogenology	2003	392	19.6	De Rensis and Scaramuzzi [70]
4	Major advances associated with environmental effects on dairy cattle	Journal of Dairy Science	2006	336	19.76	Collier et al. [71]
5	Comparison of ovarian function and circulating steroids in estrous cycles of Holstein heifers and lactating cows	Journal of Dairy Science	2004	295	15.53	Sartori et al. [72]
6	Is the temperature–humidity index the best indicator of heat stress in lactating dairy cows in a subtropical environment?	Journal of Dairy Science	2009	278	19.86	Dikmen and Hansen [73]
7	The relationship of the temperature–humidity index with milk production of dairy cows in a Mediterranean climate	Animal Research	2002	247	11.76	Bouraoui et al. [74]
8	Invited review: Effects of heat stress on dairy cattle welfare	Journal of Dairy Science	2017	240	40	Polsky and Von Keyserlink [75]
9	Effects of hot, humid weather on milk temperature, dry matter intake and milk yield of lactating dairy cows	Journal of Dairy Science	2003	229	11.45	West et al. [76]
10	Factors affecting conception rate after artificial insemination and pregnancy loss in lactating dairy cows	Animal Reproduction Science	2004	215	11.32	Chebel et al. [77]

### 3.5. Cluster Analysis of Keywords and Titles of Countries

**Figure 5:**
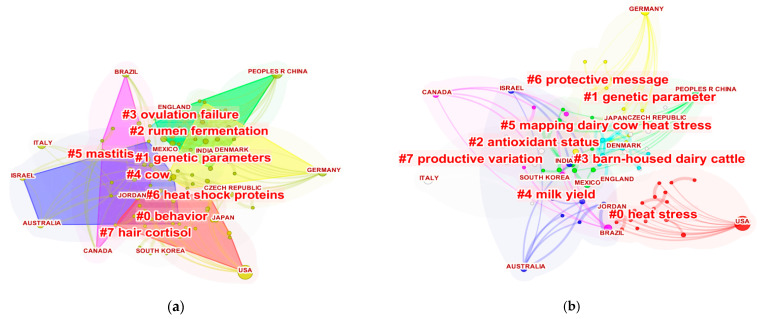
(**a**) Country grouping with keywords and (**b**) titles of the 604 studies included in the review. Identification #0 represents the grouping with the highest weight and most current area.

The countries’ interpretation was further made using the clustering of keywords, shown in Figure 5a, and title, shown in Figure 5b, with the numerical values in Tables S3 and S4. In the indexed clusters, keywords, and title 8 groups were found in each item. The clusters are formed according to weight, member number and silhouette value [33]. The keywords and title clusters showed silhouettes ranging from 0.812 to 1.000, representing good reliability of the results found [64]. In addition, the oldest study areas in the clusters presented the term # with the highest number indexing [62]. Thus, the keyword cluster #7, hair cortisol, and the title cluster #7, production variation, are the oldest areas of study. In contrast, #0 behavior and #0 heat stress are the most current areas in our research.

#### 3.5.1. Cluster Analysis of Keyword Clusters

Regarding the clusters (Figure 5a), the largest group (#0) was labeled “behavior” and had 19 members. Behavioral responses are one of the main reactions observed in animals subjected to heat stress. In a study conducted in Spain with Holstein-Friesian cows managed in a free stall system, Ramón-Moragues et al. [78] observed that heat stress (THI = 73.97) decreased the feeding (0.5 min/h), rumination (0.5 min/h) and resting times of lactating cows (2.5 min/h). Lying is a comfort behavior and a vital welfare indicator, cows in confined systems lie down an average of 10 to 12 h per day [79]. Endres and Barberg [43], when studying the cows’ behavior in a compost barn, observed an average total lying rest time for lactating cows of 12.7 h/day when THI was less than 72. In contrast, when THI was higher than 72, the cows decreased the time spent lying down (7.9 h/day) and increased the time spent standing. Increased standing time aids in body heat loss [26,80]. Furthermore, as the environment becomes more challenging (increase in the THI), cows increase the number of steps [81], water consumption [82] and agonistic behaviors [53]. 

The second most extensive set (#1) has 17 members and was labelled genetic parameter. In recent years, the effect of heat stress on genetic parameters in dairy cows has gained the focus of study due to the sensitivity of high-producing cows to heat stress and the impacts of climate change on the dairy industry [83]. In the face of constant climate change, it becomes necessary to consider the selection of cows of the Holsteins breeds more adapted to environmental conditions. However, this selection results in less productive animals [84] and lower contents in milk components, such as fat (−0.004) and protein (−0.005) in % per day [85]. 

The third largest cluster (#2) has 15 members and was labelled rumen fermentation. As mentioned previously, heat stress reduces dry matter intake (DMI) and impacts rumination in dairy cows [78]. Reduced DMI by lactating cows results in lower body heat production [86]. However, the reduction in DMI (between 28 and 34%) caused by heat stress [87] limits the number of nutrients in the mammary gland [10] and consequently reduces milk production by 25% [88] to 40% [14]. 

The 4th largest cluster (#3) has 10 members and was labelled ovulation failure. Heat stress negatively influences follicular development. Furthermore, heat stress directly impacts oocyte development from primary to secondary follicles. Oocytes have critical roles in controlling follicular granulosa cell development from the beginning of follicular organization to ovulation [89]. 

The 5th most extensive set (#4) has 10 members and was labelled cow. The 6th most extensive set (#5) has 10 members and was labelled mastitis. Among the adverse effects, heat stress also acts in limiting the immune capacity of dairy cows [90,91], which contributes to increasing milk somatic cell count (SCC) [92]. In a study conducted in Egypt to evaluate the effect of THI on milk quality in dairy cows, Nasr and El-Tarabany [93] observed a 36% increase in SCC when THI was from lowest to highest value (THI < 70 = SCC 190 cell/mL; THI 70 < 80 = SCC 216 cell/mL; THI 80–85 = 259 cell/mL).

The 7th largest cluster (#6) was labelled heat shock proteins with 9 members. Prolonged exposure of cows to heat stress damages cellular balance, thermodynamic stability, and protein synthesis [12]. However, these factors do not occur for heat-shock proteins (e.g., HSP70) [94]. HSPs play a key role in heat stress responses [95]. When a susceptible stimulus triggers a heat stress response, HSPs play a vital role in cellular thermotolerance [96,97]. However, caution is required in extrapolating the HSPs evaluation in cows since the HSPs remain constant after cows’ exposure to heat stress.

The 8th largest cluster (#7) has 8 members and was labelled hair cortisol. Heat stress plays activation of the hypothalamic–pituitary–adrenal axis in addition to increased cortisol levels in dairy cows [8]. Because cortisol rises when cows are stressed, it is considered a good indicator of animal welfare [7]. Cortisol is the first hormone observed in animals’ blood, saliva and hair when exposed to stressful situations, such as heat stress [90]. 

#### 3.5.2. Clustering Analysis of the Titles

Regarding the title cluster (Figure 5b), the largest group (#0) has 19 members and was labeled heat stress. Cluster #0 is entirely connected to all the other clusters. Heat stress impacts the entire metabolism of dairy cows. Adverse consequences are observed from short-term (up to 5 days) to long-term (greater than five days) [98], compromising productive potential [92,99,100], reproductive [101], health, oxidative balance [102], animal welfare [75] and the performance of future offspring [103]. The second most extensive set (#1) has 17 members and a silhouette value of 0.829. It is labelled as a genetic parameter. 

The 3rd largest cluster (#2) has 15 members and was labelled as antioxidant status. Oxidative stress is caused by an imbalance between antioxidants and oxidative molecules [104]. During heat stress, serum oxidative and antioxidant indices are elevated, and milk production is reduced [105]. 

The 4th largest cluster (#3) has 10 members and has been labelled barn-housed dairy cattle. Traditionally, confinement systems for dairy cows consisted mainly of tie-stalls, accessible stalls, and loose housing [106]. However, the compost barn confinement system has recently been attracting worldwide attention and demand from producers [40]. All these housing systems have their peculiarities. However, to improve the comfort of dairy cows in these systems, it is essential to prevent excess heat from entering the confinements [107]. Therefore, some criteria are fundamental to minimize this excess. The first criterion to be considered is the orientation of the barn. The east–west direction prevents the animals from being crowded in specific areas of the house due to reduced sun exposure in the morning and afternoon [108]. In addition, the roof receives a large amount of radiation that can be transmitted to the interior of the house [109] and thus, the use of thermal insulation aids in blocking radiation [65]. Another critical parameter in housing design is adequate ventilation. A proper ventilation system should contribute to a less stressful environment, with good heat and moisture renewal [110] and quality fresh air [111]. Confinement systems can feature natural ventilation [112,113], mechanical ventilation [114], ceiling fans [115], axial fans [52] and negative pressure ventilation [116,117]. The ventilation system must be activated before the heat load impacts the physiology of dairy cows. Pinto et al. [118] observed that the heat load influenced the physiological parameters when the cows were exposed to an environment with THI above 65. Thus, combining an evaporative cooling system (e.g., pads, misters, and sprinklers) and fans effectively decrease dairy cows’ heat stress in a confined system [65].

The 5th largest cluster (#4) has 10 members and was labelled milk yield. The 8th largest cluster (#7) was labelled productive variation with 8 members. Daily milk yield is the primary concern during heat stress. Heat stress alters milk yield and milk composition. The decrease in milk production is observed from three [119] to five days after exposure to heat stress [60]. Skibiel et al. [120], when analyzing the effect of heat stress in dairy cows housed in a free-stall with and without access to the cooling system, reported a decrease in milk production of approximately 5 kg/day. Further, regarding the milk decrease, Skibiel et al. [120] reported a decreased synthesis of fat (0.20 kg/day) and protein (0.10 kg/day) for cows under heat stress. Additionally, Laporta et al. [121], when analyzing (range: 2008–2018) dry Holstein cows in a free-stall with and without access to heat abatement (ventilation and sprinkler), highlighted those dry cows, when exposed to heat stress, compromised subsequent lactation up to 21%. In addition, Laporta et al. [121] identified that heat stress can compromise milk production until the third lactation and reduce the offspring’s survivability. The impacts of heat stress cause considerable economic losses. In the US, studies point to annual losses of USD 897 to USD 1.500 million for the dairy industry [122]. In another study, losses of USD 1.405 billion were observed due to climatic conditions [123].

### 3.6. Keyword Co-Occurrence Analysis

**Figure 6:**
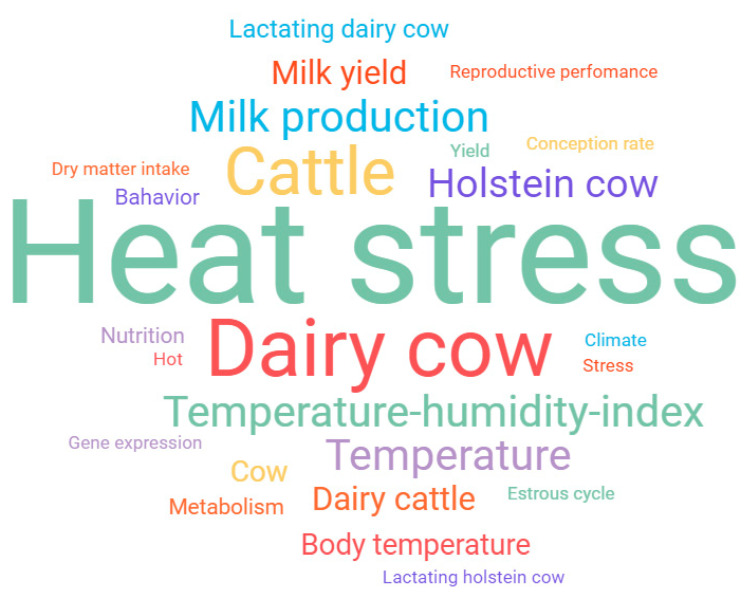
Word cloud was generated using the 25 most frequently used keywords from the 604 studies included in the review. Words appearing in more significant types were used more regularly.

The keywords most used by researchers in this line of research are presented in Figure 6; the generated network obtained 528 nodes and 3.527 links. The modularity index score (Q) was 0.5357, and the average silhouette metric (S) was 0.8783, which represented good reliability of the results found [64]. Considering that the present study aimed to perform a scientometric analysis on heat stress in dairy cows in a confinement system, it was already expected that the term “heat stress” (407) would be among the main keywords. The second most frequent keyword was a dairy cow (216), followed by cattle (169), temperature-humidity-index (103), milk production (100), temperature (100), Holstein cow (97), dairy cattle (67), milk yield (67) and body temperature (60). 

#### Clustering of Keyword Clusters

**Figure 7:**
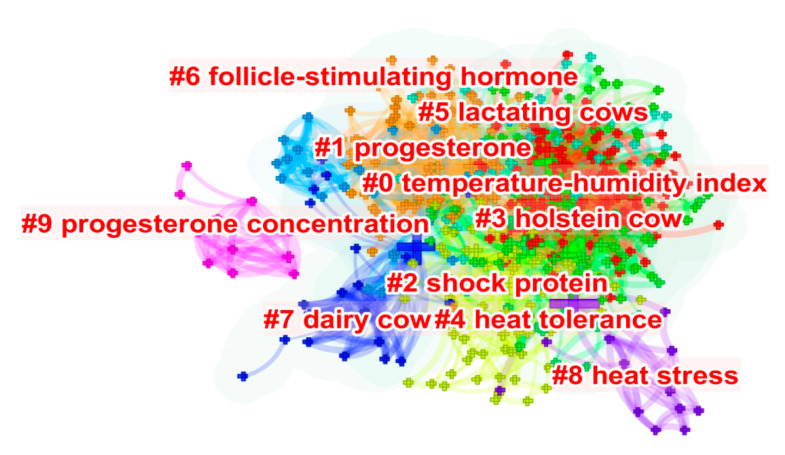
Clustering of the keywords of the 604 studies included in the review. Identification #0 represents the grouping with the highest weight and most current area.

The clustering of the keywords can be seen in Figure 7. Ten clusters were identified. The temperature and humidity index (THI) has been widely used to assess the degree of heat stress in lactating cows [92]. Several studies have reported THI values [88,124,125,126]. Physiological reactions were observed with THI higher than 65 [121]. Productive reductions were reported with THI higher than 73 [127]. Reproductive indices, such as loss in pregnancy rate (50% and 60%), were observed with THI greater than 72 [128]. Reproduction is strongly impacted by heat stress. Cows exposed to heat stress alter progesterone levels, compromising fertility. Thus, follicular development is impaired, leading to abnormal oocyte maturation and embryonic death [70]. These impacts are mainly witnessed in high-producing cows, such as the Dutch breed [23]. However, heat-stressed cows exhibit increased levels of HSP70 (from 13.66 ng/mL to 22.99 ng/mL) [129] to aid in body thermoregulation [96,97]. Thus, HSP70 may be a biomarker for identifying heat stress in lactating cows [129,130]. Table S4 shows the numerical information of each keyword cluster.

### 3.7. Analysis of Journals, Authors and Institutions

**Figure 8:**
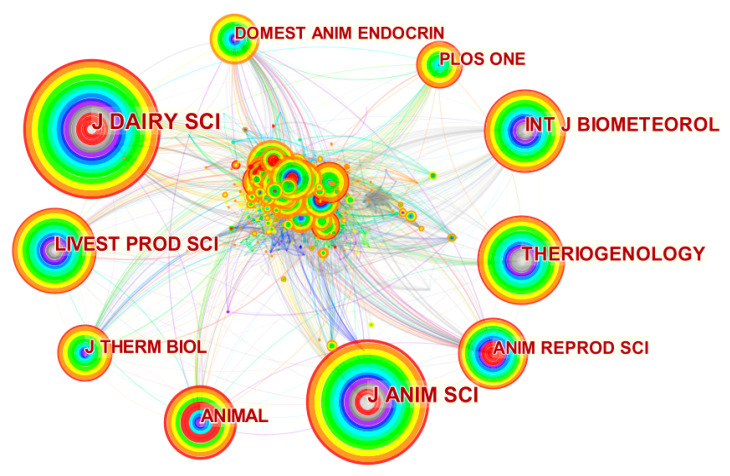
Co-occurrence network of the journals of the 604 studies included in the review. The node size is determined by the number of times this word was used and the links in the relationship between the journals. Warm colors (red, yellow, and green) represent the most current studies, while cold colors (gray, dark blue and light blue) represent the oldest studies.

Contributing journals on heat stress in lactating cows in confinement systems were analyzed. The generated network identified the high-impact journals. The developed network contained 678 nodes and 8.095 links. The 10 journals with the most significant nodes are presented in Figure 8. 

The ranking of the journals about frequency and impact factor by Journal Citation Reports (JCR) are shown in Table 2. Within this theme, the node size is represented by the frequency of publications, while the burst is by the number of citations illustrated in the figure by the red center [33].

The Journal of Dairy Science stands out as the science center with the highest frequency (539). In addition, it had a burst of 5.04, indicating that this journal has essential studies for the scientific community. This journal has the highest impact factor in this area of research (4.034). The impact factor is a metric created in 1961 by Garfield and Sher to help researchers choose journals [131]. The impact factor shows the average number of citations in a journal over the past two years [132]. Furthermore, this journal contains 6 of the 10 most cited articles related to heat stress in the confinement system for dairy cows (Table 1), which confirms the importance of this journal in this study area. 

The second most crucial journal in this line of research was the Journal of Animal Science, which presented a frequency of 426. Moreover, this journal showed that burst (3.18) and its impact factor (3.159) are high for the animal area. On the other hand, the journal Animal Reproduction showed the most elevated burst (7.06), demonstrating that the articles published in this journal were highly cited in each period [33]. In addition, the Journal of Dairy Science, Journal of Animal Science and Animal Reproduction show cool and warm colors representing that these journals have both old and current articles [33].

Based on the number of articles, the 10 most productive authors were found according to the WoS database, as shown in Figure S1. Of the 554 authors identified, researchers L. H. Baumgard and R. J. Collier were the most productive authors. In addition, the 10 most productive institutions were found (Figure S2). The Chinese Academy of Agricultural Sciences, followed by the State University System of Florida and the University of Florida, were the institutions that presented the highest number of published studies. The authors and institutions are entirely linked with the countries with the most publications (Figure 4).

### 3.8. Limitations of the Study

Scientometrics allows one to gather various information across studies, summarizing and listing future trends. Although this analysis synthesizes important information across studies, it has some limitations. First, studies on heat stress in lactating dairy cows in the confinement system may have yet to be located from our search strategy using Topic Search in WoS. Although WoS is considered one of the most reliable and comprehensive databases of scientific publications, relevant results may have yet to be found. Second, the search term used may have yet to see some studies, so this study is susceptible to using keywords in searching for publications from the title, abstract and keywords. Third, the study was limited from 2000 to April 2022. Thus, some critical studies may need to be considered. Fourth, the data extracted in the WoS database may have needed to be standardized across studies and, therefore, may have contained an error, such as duplicates and erroneous entries.

## 4. Conclusions

This review provides a comprehensive scientometric investigation of heat stress in lactating cows in confined systems, providing the study of the art and future perspectives. The US was the publication leader, followed by China, Italy and Germany. Studies in nutrition, reproduction, production, genetics, immunology, behavior, animal physiology, thermal environment and types of confinement were the most reported areas. The term’s behavior, temperature and humidity index, and heat stress are the trends in this study. However, scientometric analysis can predict short-term predictions in this research field; thus, long-term predictions should be avoided. However, it is worth mentioning that the compost-bedded pack barn system is relatively new, and studies evaluating heat stress in this housing system are extremely necessary. More studies evaluating heat stress in lactating cows in confinement systems are needed. The results of this review may interest researchers in this area of study in their future studies.

## Figures and Tables

**Table 2:** Ranking scientific journals concerning frequency and impact factor by Journal Citation Reports (JCR) in 2021.

Journal	Frequency	Burst	Half-Life	Year	Impact Factor 2021
Journal of Dairy Science	539	5.04	17.5	2000	4.225
Journal of Animal Science	426	3.18	17.5	2000	3.338
Livestock Production Science	249	NA	17.5	2000	1.929
International Journal of Biometeorology	241	NA	18.5	2000	3.738
Theriogenology Animal Reproduction	235	NA	16.5	2001	2.923
Animal	203	NA	6.5	2012	3.730
Animal Reproduction	138	7.06	16.5	2001	2.220
Plos One	125	NA	4.5	2015	3.240
Domestic Animal Endocrinology	110	NA	14,5	2004	2.290

NA = Not applicable.

## Data Availability

Not applicable.

## Supplementary Materials

The following supporting information can be downloaded at: https://www.mdpi.com/article/10.3390/ani13030350/s1, Figure S1: Ranking of the ten authors producing knowledge about heat stress in lactating cows in a confinement system; Figure S2: Ranking of the ten institutions producing knowledge about heat stress in lactating cows in a confinement system; Table S1: Top 13 countries in terms of publication; Table S2: Sizes and silhouettes of the keyword clusters; Table S3: Sizes and silhouettes of the title clusters; Table S4: Keyword Clusters Summary.
