# Supplementary material for: Effects of Heat Stress in Dairy Cows Raised in the Confined System: A Scientometric Review
Source: Animals (Basel). 2023 Jan 19;13(3):350. doi: 10.3390/ani13030350 (PMC9913584; doi:10.3390/ani13030350)
Supplement: Supplementary file 1 [file animals-13-00350-s001.zip › animals-2070859-supplementary.pdf]

Figure S1. Ranking of the ten authors producing knowledge about heat stress in lactating cows in a confinement system.

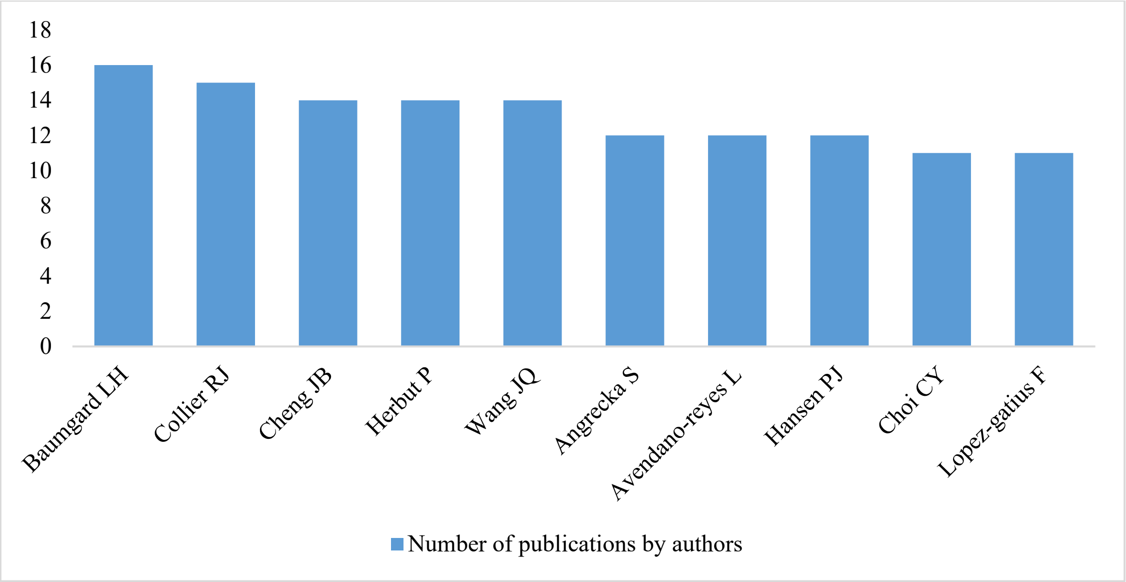

Figure S2. Ranking of the ten institutions producing knowledge about heat stress in lactating cows in a confinement system

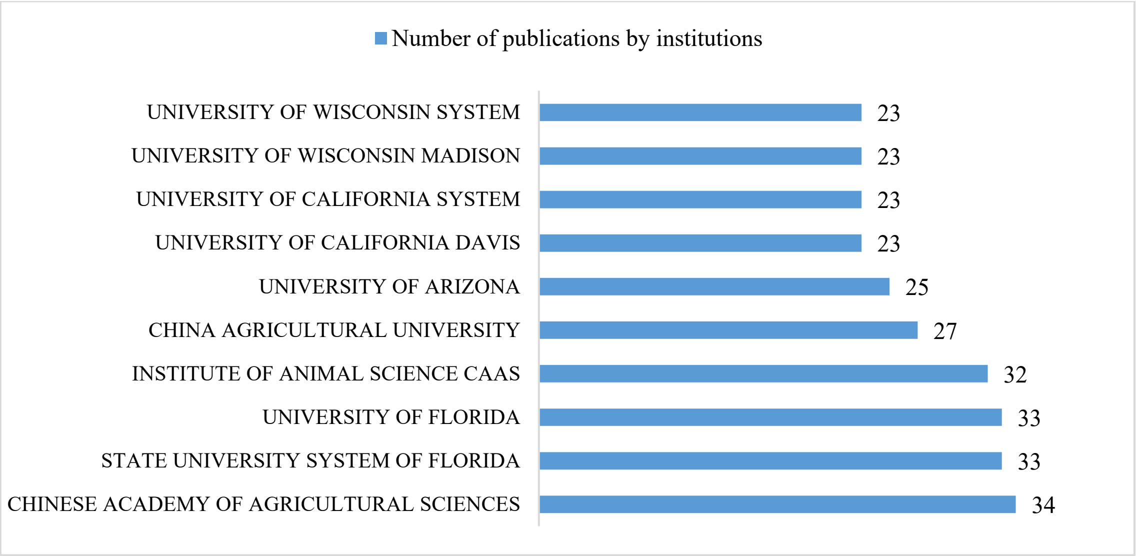

Table S1.Top 13 countries in terms of publication

Country	Frequency	Explosion	Half-life	Year
USA	187	8.00	16.5	2001
China	85	NA	10.5	2008
Italy	46	NA	13.5	2003
Germany	43	3.61	11.5	2007
Brazil	37	NA	15.5	2002
Israel	26	3.83	13.5	2006
Canada	26	NA	16.5	2002
Australia	26	NA	13.5	2006
Mexico	21	NA	13.5	2003
Japan	18	NA	11.5	2006

Spain	18	NA	11.5	2007
India	18	NA	11.5	2007
Poland	17	NA	12.5	2005

NA= Not applicable

Table S2. Sizes and silhouettes of the keyword clusters

Cluster ID	Size	Silhouette	Year	Cluster label
0	19	1	2010	Behaviour
1	17	0.829	2013	Genetic parameter
2	15	0.838	2014	Rumen fermentation
3	10	0.918	2014	Ovulation failure
4	10	0.830	2010	Cow
5	10	0.898	2012	Mastitis
6	9	0.829	2011	Heat shock proteins
7	8	0.812	2015	Hair cortisol

Table S3. Sizes and silhouettes of the title clusters

Cluster ID	Size	Silhouette	Year	Cluster label
0	19	1	2010	Heat stress
1	17	0.829	2013	Genetic parameter
2	15	0.838	2014	Antioxidant status
3	10	0.918	2014	Barn-housed dairy cattle
4	10	0.830	2010	Milk yield
5	10	0.898	2012	Mapping dairy cow heat stress
6	9	0.829	2011	Protective message
7	8	0.812	2015	Productive variation

Table S4. Keyword Clusters Summary

Cluster ID	Size	Silhouette	Year	Cluster label	Alternate label
0	118	0.649	2013	Temperature-humidity index	heat stress; dairy cattle; milk production; milk yield; milking performance; dairy cow; lactating dairy cow; holstein cow; heat stress condition; physiological responses
1	83	0.604	2009	Progesterone	heat stress; dairy cow; lactating dairy cow; dairy cattle; artificial insemination; heat-stressed dairy cattle; blood flow; ovsynch protocol; corpus luteum; dairy cattle welfare
2	82	0.615	2017	Shock protein	heat stress; dairy cow; lactating dairy cow; holstein cow; milk yield; milk production; heat-stressed lactating holstein cow; body temperature; heat-stressed dairy cow; short communication
3	70	0.690	2009	Holstein cow	dairy cow; heat stress; lactating dairy cow; milk production; milk yield; production responses; physiological indicator; production

					performance; lactating cow; dry matter intake
4	48	0.763	2011	Heat tolerance	heat stress; dairy cow; holstein cow; milk yield; lactating dairy cow; reproductive performance; high producing dairy cow; southern apennine; ventilation effects performance
5	43	0.821	2008	Lactating cows	heat stress; dairy cow; lactating dairy cow; dairy cattle; milk production; temperature-humidity index; dairy farm; thermal stress; ambient temperature; acute heat stress
6	25	0.916	2006	Follicle-stimulating hormone	dairy cow; heat-stressed cow; bovine somatotropin; inflammatory response; dairy cattle; heat stress; holstein cow; oocyte competence; load indice; conception rate
7	24	0.943	2011	Dairy cow	dairy cow; heat stress; lactating dairy cow; milk production; holstein cow; body temperature; milk yield; reproductive performance; core body temperature
8	83	0.978	2005	Heat stress	heat stress; dairy cow; lactating dairy cow; heat-stressed lactating holstein cow; milk yield; holstein cow; dairy cattle; reproductive performance; temperature-humidity index
9	11	0.946	2004	Progesterone concentration	hot environment; dairy cow; lymphocyte function; following superovulatory treatment; progesterone profile; progesterone profile; tropical environment; holstein-friesian dairy cow; oestrous cycle change; following superovulatory treatment
